# Evaluating the Accuracy of Declared Eating Schedules by Continuous Glucose Monitoring

**DOI:** 10.3390/nu18050772

**Published:** 2026-02-27

**Authors:** Pedro González-Romero, Juan Antonio Madrid, Pedro Francisco Almaida-Pagán, Maria Angeles Rol

**Affiliations:** 1 Chronobiology and Sleep Laboratory, Department of Physiology, University of Murcia, Mare Nostrum Campus, IUIE, 30100 Murcia, Spain; jamadrid@um.es (J.A.M.); pfalmaida@um.es (P.F.A.-P.); angerol@um.es (M.A.R.); 2 Biomedical Research Institute of Murcia Pascual Parrilla—IMIB, 30120 Murcia, Spain; 3 Ciber Fragilidad y Envejecimiento Saludable (CIBERFES), Instituto de Salud Carlos III, 28029 Madrid, Spain

**Keywords:** chrononutrition, circadian rhythms, glucose monitoring

## Abstract

**Background/Objectives**: Chrononutrition is an emergent field concerning the effect of eating patterns on human health and their relationship with biological rhythms. Current evidence points towards the benefits of early eating in the prevention of non-communicable diseases and circadian health. Despite the importance of eating/fasting rhythm, current methods are neither specific nor validated against physiological variables. This work aimed to explore an objective metabolic outcome, postprandial glucose, as an accuracy indicator of self-declared meal schedules registered in a mobile app. **Methods**: A 1-week protocol of ambulatory monitoring of meal schedules, glucose, and circadian variables was performed in 20 young adults. Meal annotations were registered using KronoEat 1.0, a smartphone app, allowing for both prospective and recall entries. A circadian monitoring device provided data on movement intensity, distal skin temperature, and prospective food annotation. **Results**: Participants annotated an average of 3.5 food events/day/participant with KronoEat. Breakfast (92.7%) and lunch (86.4%) showed the highest proportion of food events related to a glycemic excursion, whereas this proportion was lower for dinner (79.7%) and snacks (67.7%). Postprandial glucose after main meals differed significantly from average glucose levels. Interesting couplings were found in circadian variables and glucose—for example, between post-breakfast glycemic excursions and the morning increase in activity. **Conclusions**: Meal schedules registered under free-living conditions in KronoEat show high levels of correlation with postprandial glucose and glycemic excursions derived from continuous glucose monitoring.

## 1. Introduction

Nutrition research has historically focused on the quantity and content of food. However, the recent literature highlights the importance of eating schedules to human health [1]. In this context, chrononutrition has emerged as an integrative field, exploring how the timing of food intake can be useful in preventing and managing health issues.

The impact of eating schedules on human health is modulated by the circadian system, a complex network of molecular clocks [2]. The central pacemaker is located in the suprachiasmatic nuclei (SCN) of the hypothalamus, which are entrained mainly by the environmental light–dark cycle [3]. SCN neurons show a characteristic autonomous oscillation of around 24 h, coordinating key physiological processes such as the sleep–wake cycle, body temperature and cognitive state [4]. Importantly, other body tissues have peripheral clocks, which are synchronized by the SCN through humoral, physiological and neural signals [5].

Circadian oscillation of metabolic factors is thought to increase daily energy expenditure efficiency and to separate in time conflicting biochemical routes. Key metabolic processes such as glucose uptake by the liver, fatty acid synthesis, gluconeogenesis and ammonia production are under circadian regulation [6]. Consistently, glucose tolerance and the thermic effect of food exhibit a daily rhythm under circadian control, with higher values during the daytime [7]. Therefore, eating during the day and fasting during the night seem to be optimal for metabolic health.

Fasting routines promote beneficial processes at the cellular level [8]. After hours without eating, the human body starts using lipids to produce energy. In addition, ketone bodies, which are subproducts of lipids catabolism, are also signaling molecules capable of enhancing DNA repair, autophagy, mitochondrial biogenesis, and reducing inflammation [9]. However, fasting duration is not the only factor that affects human metabolism; a late eating window can delay and reduce this process during the night [10]. In the same direction, later lunch times have been associated with less weight loss during dieting [11] and higher body fat percentages [12]. Furthermore, early meal patterns have the potential to prevent and manage metabolic disturbances. For instance, routines promoting shorter and earlier eating windows are capable of improving glucose homeostasis traits, such as insulin sensitivity [13] and daily average glucose [14].

Meal timing is also closely related to sleep health. Most evidence indicates that a delayed eating windows is associated with poorer sleep quality [15]. In fact, being a late eater is associated with lower sleep efficiency [16] and more nocturnal awakenings [17].

The detrimental effects of late eating windows might be partly driven by the differential entrainment of SCN and peripheral clocks. While eating time has a slight synchronizing effect on the SCN [18], liver cells, for instance, are substantially entrained by food intake timing [19]. Indeed, hepatocytes do not depend on the SCN to maintain the circadian component of their genetic expression [20]. Consequently, irregular or delayed eating patterns have the potential to uncouple the coordinated oscillation of central and peripheral clocks, leading to circadian misalignment [21]. For example, a mismatch in eating schedules between workdays and free days (social jet lag) has been associated with higher body mass index [22]. A more extreme case of chronodisruption is found in shift workers, whose external cues are forced to be misaligned with the internal time dictated by the SCN, showing an increased risk of developing obesity, cardiovascular diseases, and diabetes [23].

In this context, there is a growing need to develop standardized, feasible and accurate methods to monitor and evaluate eating schedules under free-living conditions. In fact, most existing approaches are not specifically designed for eating time evaluation but for nutritional assessment. Among them, 24 h dietary recalls are widely used and have been adapted to include temporal information, but they are prone to recall bias [24]. Although some technological attempts to obtain passive measures have been implemented, they require sensors that can be uncomfortable to wear over long periods [25] and are not sufficiently accurate [26].

Moreover, although blood glucose levels are known to be influenced by physical activity and sleep disturbances [27,28], few studies have simultaneously evaluated eating time, circadian variables, and metabolic outcomes under real-life conditions. In this sense, ambulatory circadian monitoring, using a wrist-worn device, is a non-invasive tool that allows for multivariable recording, allowing for the integration of meal timing with circadian-related variables, including distal skin temperature, activity and sleep [29]. This technology has been validated to determine sleep periods [30] and has been used in previous studies to evaluate sleep alterations [31]; thus, it constitutes a useful approach when combined with glucose monitoring, with minimal subject disturbance.

Self-declared annotations of daily eating times, combining both prospective and recall data, might be a useful alternative means of characterizing properly eating schedules, but one of the main problems in chrononutrition is the lack of validation of meal time entries against objective physiological indicators. Due to its postprandial dynamics, interstitial glucose measured by continuous glucose monitoring (CGM) could be a relevant marker of meal timing accuracy.

Thus, this study proposes that self-declared meal schedules, registered with a smartphone application, are physiologically accurate when temporally coupled with CGM-derived postprandial glucose and glycemic excursions. Additionally, the relationships between circadian variables—assessed by ambulatory circadian monitoring—and postprandial glucose levels are explored.

## 2. Materials and Methods

### 2.1. Experimental Design

Twenty young and healthy university students (18–24 years old; 55% male; BMI: 23.25 ± 0.75) were included in this study after satisfying the following criteria: (1) normal color vision; (2) absence of chronic diseases and pharmacological treatments; and (3) no history of shift work or transmeridian travels during the three months before the study. The sample size was chosen in accordance with previous exploratory approaches in CGM [32,33].

**Figure 1:**
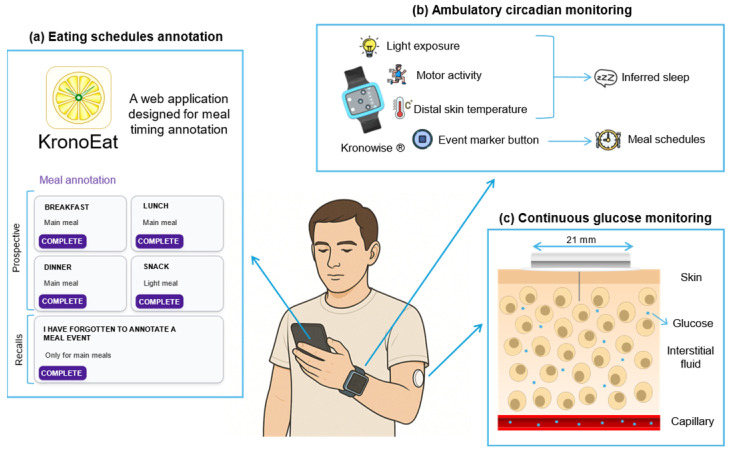
A representative scheme of the experimental procedure of the 1-week protocol. (**a**) Interface of KronoEat 1.0, an application designed for research purposes in the field of chrononutrition. Prospective and recall eating time patterns are easily recorded by the volunteer using their respective buttons. KronoEat was developed in Spanish, so the interface is shown translated into English. (**b**) Different variables measured with Kronowise 3.0^®^ (Kronohealth SL, Murcia, Spain), an ambulatory circadian monitoring device. It includes the possibilities of estimating sleep and annotating prospectively meal events. (**c**) Details of continuous glucose monitoring functioning at tissue scale. The sensor FreeStyle Libre 3^®^ (Abbot Laboratories, Abbott Park, IL, USA), attached to the arm skin, measures glucose concentration in interstitial fluid with a thin filament.

Volunteers were enrolled in a simultaneous one-week protocol of continuous glucose monitoring (CGM), ambulatory circadian monitoring (ACM) and eating time annotation (Figure 1). During the protocol, volunteers maintained their normal daily routine.

Participants signed an informed consent document. Procedures were authorized by the Ethics Committee of the University of Murcia (M10/2024/099) and followed the ethical principles of the Declaration of Helsinki.

### 2.2. Continuous Glucose Monitoring (CGM)

Interstitial glucose, a reliable marker of blood glucose levels, was measured with the commercially available and validated sensor FreeStyle Libre *3*^®^ (Abbot Laboratories, Abbott Park, IL, USA) [34]. The device was placed in the participants’ non-dominant arm, leaving a thin filament with an electrochemical sensor under the skin, which continuously measures glucose levels.

The sensor measures glucose once per minute (0.017 Hz), sending this metabolite data to a smartphone application for sensor users (Libre 3) via Bluetooth^®^. Participant profiles were linked with LibreView, a professional web interface for researchers and physicians.

Although sensors are manufactured to function for 14 days, only the first 7 days of monitoring were considered for this study, allowing for the coupling of ACM and CGM data. Glucose datasets for each participant were obtained in CSV format from LibreView, averaged into 5 min epochs. Although a minimum of 3 complete days of glucose monitoring was considered acceptable for inclusion, most participants (90%) had 7-day recordings. Two participants experienced missing data, one because of a CGM sensor error and the other because of an accidental sensor detachment.

### 2.3. Ambulatory Circadian Monitoring (ACM)

Participants wore the Kronowise 3.0^®^ device (KW; Kronohealth SL, Murcia, Spain) on their non-dominant wrist for 7 consecutive days under real-life conditions. KW is a compact wristwatch-like device equipped with several sensors that continuously record the following circadian variables: (1) distal skin temperature (DST) as a proxy circadian marker; (2) triaxial motor activity; and (3) light exposure in three spectral bands (total light, 460–490 nm; blue light, >800 nm; infrared light). KW also includes an event marker.

Circadian-related data were collected at a frequency of 10 Hz for motor activity and 1 Hz for DST and light exposure. Although several motor activity variables can be derived from the MEMS (MicroElectroMechanical Systems)-calibrated triaxial accelerometer integrated into the KW, only movement intensity (triaxial acceleration; MI) was considered in this study. Briefly, MI was calculated as the sum of the absolute values of the acceleration vectors (for more details see [30,35]). ACM data were stored in 30 s average epochs in a TXT file, directly exported from the device.

### 2.4. Meal Time Registration

The web application KronoEat (KE; Chronobiology and Sleep Laboratory, University of Murcia, Murcia, Spain; version 1.0) was specifically implemented in this study to characterize meal schedules. KE is a virtual alternative to written meal-time diaries, designed to increase experimental engagement in a society increasingly connected to smartphones. It is installed using a unique code for each participant, provided during the recruitment process.

KE allows for the annotation of both prospective and retrospective data. Prospective meal events can be introduced as (1) breakfast, (2) lunch, (3) dinner and (4) snack. Times and dates of meal events are automatically synchronized with the smartphone clock. Retrospective annotations are restricted only to main meals, excluding snacking. In this case, time and date are manually entered by the user. Although KE was designed in Spanish, English-translated interface screenshots can be seen in Figure S1.

Participants were asked to record the time of their eating occasions using KE, prioritizing real-time annotation during eating. Additionally, they were also asked to push the central marker button of the KW device while they were eating as an additional prospective data collection method. Meal data were filtered, excluding annotations that were too close to each other (≤15 min) using the same method to avoid redundancy. When a KE annotation was close (≤30 min) to a KW event, both annotations were considered as the same eating occasion. In addition, when glucose data were missing for a particular feeding event, the corresponding KW or KE annotation was not considered in the overall analysis.

### 2.5. Protocol Accuracy

Glycemic excursions, defined as significant upward fluctuations in glucose levels recorded by CGM, were used to estimate the accuracy of meal events annotations under the following assumptions: (1) after a meal, a glucose peak typically occurs, and (2) if meals were correctly annotated, the participants’ average postprandial glucose would be significantly higher than their mean glucose.

**Figure 2:**
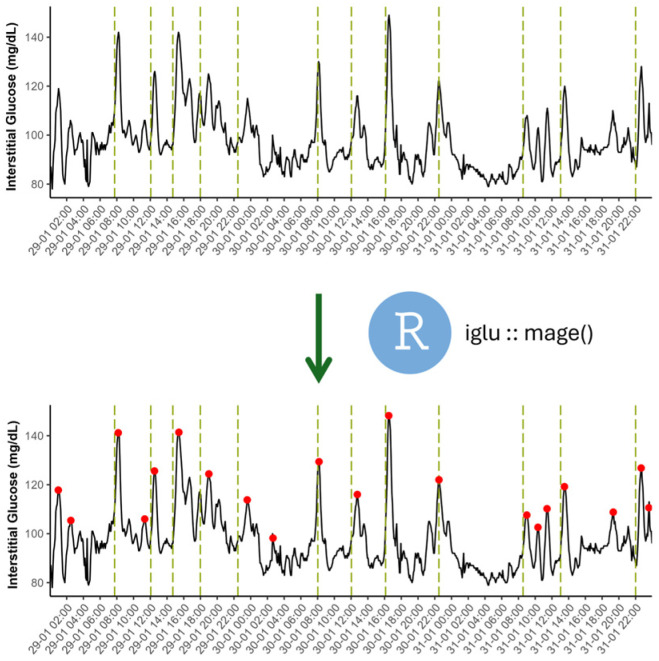
Graphical representation of glucose peak calculation using the R package iglu. This statistical tool automatically identifies the glycemic excursions of a glucose trace and measures their amplitude [36]. Meal events are shown with vertical green dotted lines, whereas glucose peak timing is indicated by a red dot. This graph corresponds to real data obtained in the present study, showing glycemic excursions both related and not related to eating events.

The calculation of the mean amplitude of glycemic excursions (MAGE) through a validated algorithm [36] enabled the identification of individual peaks in the glucose record (Figure 2). This process was mainly supported by the R package (iglu https://cran.r-project.org/package=iglu, accessed on 6 November 2025). Originally developed to analyze glycemic markers in CGM data, this tool was also useful for identifying and excluding abnormal glycemic patterns in participants. Glucose status in the present study was evaluated according to reference values reported in previous studies [37,38].

The nearest glucose peak after each KE annotation was calculated. Since our volunteers were self-reported as healthy, only peaks found in the first 2 postprandial hours were accurately associated with a food event. The ratio between food events with and without an assigned glucose excursion was defined as peak-related accuracy.

As visual support, representations of the average postprandial glucose levels and *zeitgeber* times (ZT) of glucose peaks were graphed exclusively for main caloric meals (breakfast, lunch, dinner). *Zeitgeber* time (ZT) graphs are commonly used in the field of chronobiology to study the response of certain variables to photoperiod [39]. These graphs establish the beginning of light phase as ZT = 0. In the present study, ZT = 0 was defined as the time point of each glucose peak associated with a meal event, allowing the temporal architecture of glucose responses to be analyzed separately for each meal type. CGM data was represented jointly with MI and DST data from ACM.

### 2.6. Statistical Analysis

Data was processed and analyzed with the software R 4.5.0 using the interface RStudio 2025.05.0. Values are shown as mean ± standard error of the mean. The normality of the data was checked with a Shapiro–Wilk test. As the normality of every variable could not be assured, differences were analyzed with non-parametrical tests for dependent samples: Wilcoxon signed-rank test (2 groups), or Friedman test followed by a pairwise Wilcoxon post hoc test, adjusted by Bonferroni corrections (more than 2 groups).

## 3. Results

### 3.1. Participants Glucose Status

Participants showed normal average levels of interstitial glucose (97.00 ± 1.68 mg/dL) and mean amplitude of glycemic excursions (37.91 ± 1.48 mg/dL). Their coefficients of variation (15.94 ± 0.80) and time in range (96.1 ± 1.1%) were also compatible with normal glucose homeostasis.

### 3.2. Meal Time Annotation and Peak-Related Accuracy

After 1 week of monitoring, 452 meal annotations from KW and 533 from KE were recorded. After filtering, 425 KW and 494 KE entries were included in the analysis. The annotation coincidence between KW and KE was 80.57% (398 events coupled to KW/494 total KE events).

**Figure 3:**
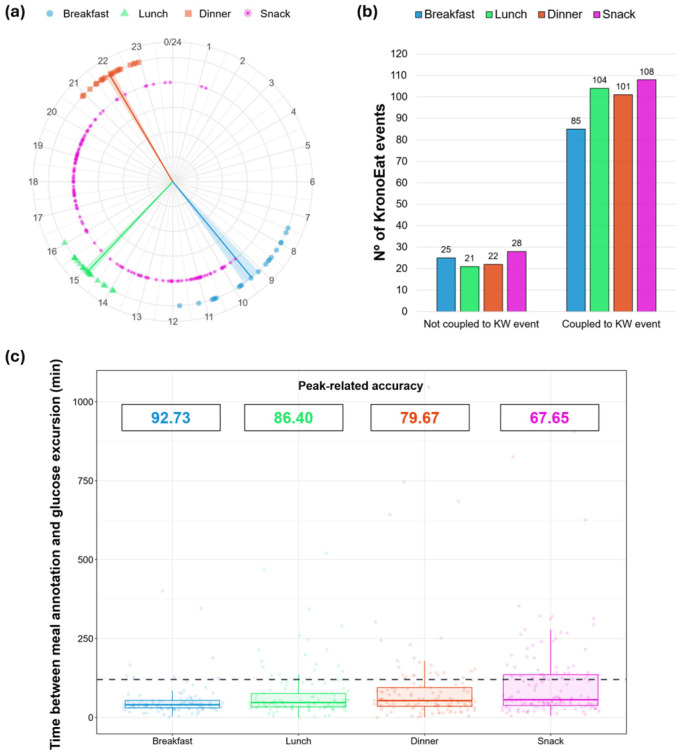
Self-reported meal schedules of participants obtained using KronoEat and their temporal relationship with glycemic excursions. (**a**) Meal schedules of participants. Mean timing values (n = 20) of breakfast (blue circles), lunch (green triangles) and dinner (red squares) are represented via a 24 h clock for each participant. Solid lines and shaded areas indicate, respectively, the mean and standard error of the mean for each meal type. All snacking occasions reported by participants (n = 136) are drawn as pink asterisks. (**b**) Number of KronoEat annotations classified by eating occasion type and their coincidence with Kronowise (KW) prospective events. (**c**) Temporal distance between each food annotation and its nearest postprandial glucose peak. A dashed line separates meal events associated with a glucose peak (≤120 min) from those not associated (>120 min). The ratio between peak-related annotations and total annotations is shown for each eating occasion as the peak-related accuracy in the upper part of the graph.

Meal types reported in KE were well-balanced, allowing for the characterization of the average main meal times and snacking occasions for every participant (Figure 3a). Mean timing values for breakfast (9.37 ± 1.28), lunch (14.90 ± 0.55) and dinner (21.96 ± 0.60) were consistent with a late eating window. Only 30 KE records (6.1%) were retrospective.

The most frequent annotated meal was the snack (27.5%), followed by lunch (25.3%), dinner (24.9%) and—the least frequent—breakfast (22.3%). In addition, the proportion of KE entries that matched with KW annotations was similar across meal types (79.4–83.2%), except for breakfast (77.3%) (Figure 3b).

The nearest glucose peak after each KE-annotated meal was considered to be linked with the eating occasion when it was found within the two postprandial hours. The ratio between meals linked with a glucose peak and total food annotations was calculated and described as peak-related accuracy (Figure 3c). This index showed higher values for breakfast (92.7%, 102/110) and lunch (86.4%, 108/125) than for dinner (79.7%, 98/123) and snacks (67.7%, 92/136).

### 3.3. Postprandial Glucose

**Figure 4:**
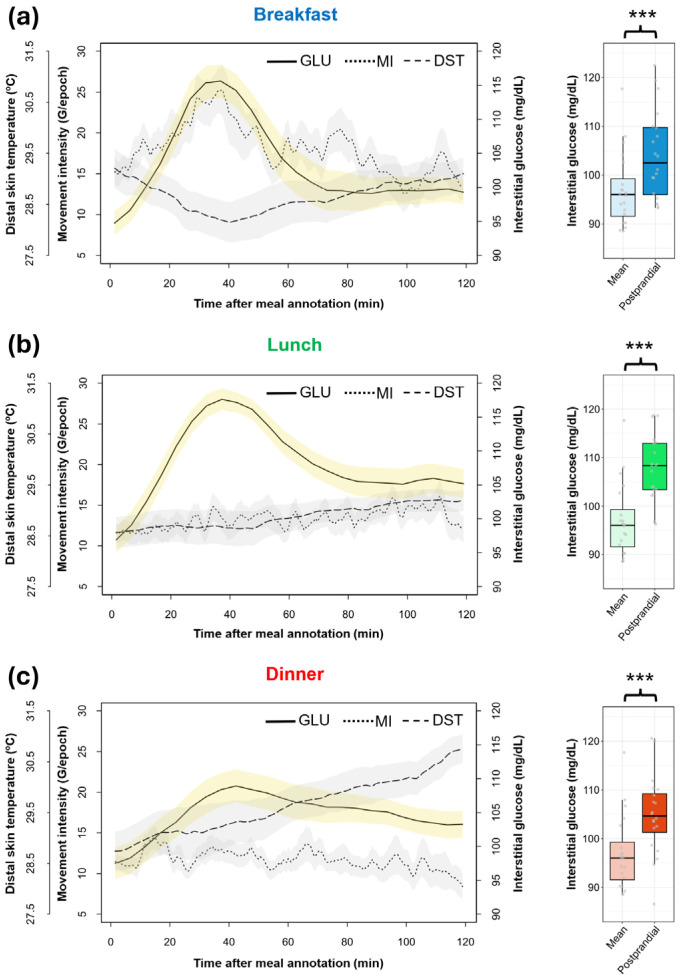
Mean 2 h postprandial glucose of participants (n = 20), considering meal schedules registered in KronoEat and continuous glucose monitoring, separated by (**a**) breakfast, (**b**) lunch and (**c**) dinner. Mean values for glucose (line) are represented alongside motor intensity (dotted line) and distal skin temperature (dashed line) obtained via ambulatory circadian monitoring. The standard error of the mean is indicated by the shaded areas. The postprandial glucose values of the volunteers are compared with their respective mean interstitial glucose values on the right side of the figure. Wilcoxon signed-rank test significance: *** *p* < 0.001. GLU: Interstitial Glucose; MI: Movement Intensity; DST: Distal Skin Temperature.

Glucose values two hours after the KE-annotated meals were evaluated for each participant. Postprandial glucose for every main meal was significantly higher compared to the average glucose of the participants, proving that the declared mealtimes of the participants were generally followed by considerable metabolic changes (Figure 4).

Graphs reveal different patterns of glucose, primarily in post-dinner levels (104.83 ± 1.81 mg/dL), showing a more flattened curve, consistent with a lower detection of glycemic excursions after the meal. Moreover, post-lunch values (107.99 ± 1.48 mg/dL) show less clearance in the second postprandial hour, compared with post-breakfast levels (104.10 ± 2.02 mg/dL). However, no differences in the mean value of postprandial glucose were found between different types of meals (*p* = 0.165). Consistently, area under the curve values of postprandial glucose after breakfast (11,927.07 ± 214.13), lunch (12,456.57 ± 170.60) and dinner (12,060.59 ± 212.06) were not significantly different (*p* = 0.086).

Circadian variables were also studied after mealtime, finding that both MI (*p* < 0.001) and DST (*p* < 0.05) mean values were different between different types of meals. Post-breakfast values (18.11 ± 0.93 G/epoch) were higher than both post-lunch (13.26 ± 0.68 G/epoch; *p* < 0.001) and post-dinner MI values (11.64 ± 0.70 G/epoch; *p* < 0.001), which can be visually appreciated in the rising curve in Figure 4a, coinciding with a glucose increase. As expected, the post-breakfast (28.66 ± 0.33 °C; *p* < 0.05) and post-lunch (28.87 ± 0.24 °C; *p* < 0.05) values of DST were significantly lower than those after dinner (29.64 ± 0.35 °C). As already described, postprandial elevation of DST after lunch is preserved, with an average increase of 0.65 °C after two postprandial hours.

### 3.4. Zeitgeber Time of Glycemic Excursions

**Figure 5:**
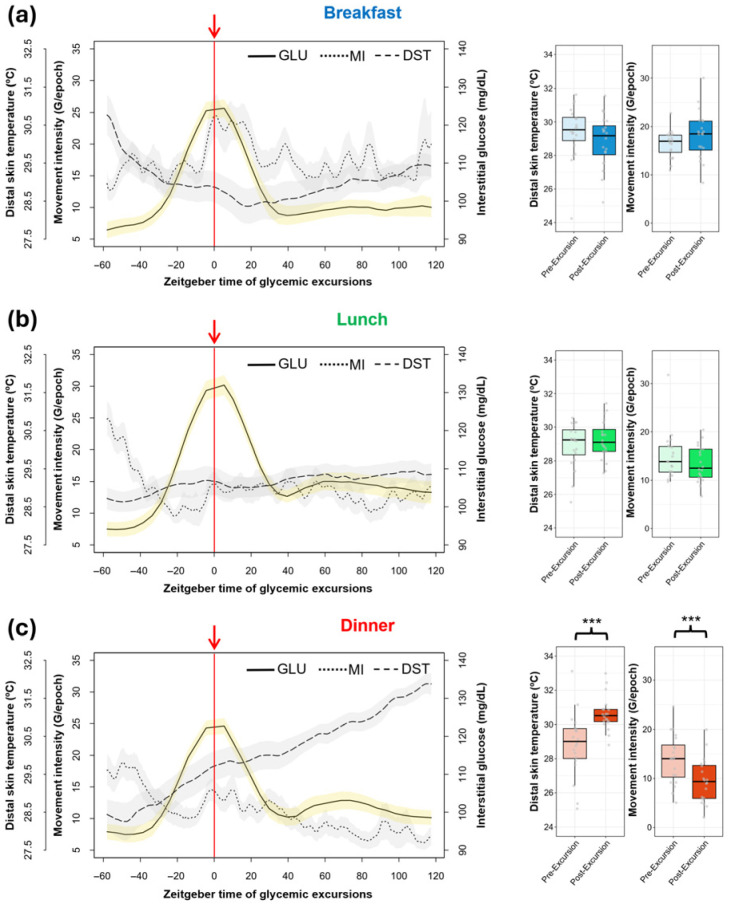
Zeitgeber time representation of glycemic excursions linked to KronoEat annotations for (**a**) breakfast, (**b**) lunch and (**c**) dinner. In addition to average glucose levels (line), the mean values of motor activity (dotted line) and distal skin temperature (dashed line) from ambulatory circadian monitoring are also shown. The standard error of the mean is indicated by the shaded areas. The red arrow and line indicate the time zero of the glucose peak. Pre-peak mean levels (1 h) of circadian variables are compared with post-peak mean levels (2 h). Wilcoxon signed-rank test significance: *** *p* < 0.001. GLU: Interstitial Glucose; MI: Movement Intensity; DST: Distal Skin Temperature.

The architecture of the glucose peaks associated with declared eating occasions in KE was revealed via zeitgeber time representations (ZT), in which “ZT = 0” corresponds to the time of glycemic excursion. Concurrently, average circadian variables (MI, DST) were analyzed (Figure 5).

The average time between meal annotation and its respective glycemic excursion was similar between different types of meals (breakfast: 41.52 ± 2.42 min; lunch: 48.37 ± 2.80 min; dinner: 46.95 ± 3.36 min; *p* = 0.157). A lower amplitude of dinner glycemic excursions can be seen in Figure 5c, and significant differences with respect to nadir-to-peak amplitude after breakfast (*p* = 0.009) and after lunch (*p* = 0.011) were found. Amplitudes of excursions after breakfast and lunch were, on average, 10 mg/dL higher than those after dinner (38.40 ± 2.54 mg/dL).

The movement intensity curve for breakfast ZT reveals an activity increase, slightly delayed from the glycemic excursion, but the activity values before (16.45 ± 0.63 G/epoch) and after (18.13 ± 1.13 G/epoch) breakfast glucose peaks did not differ (*p* = 0.231).

Pre-peak activity at lunch (14.96 ± 1.10 G/epoch) and dinner (13.89 ± 1.11 G/epoch) exhibited a decrease, contrary to the low and stable values found in post-peak activity (lunch: 13.40 ± 0.83 G/epoch; dinner: 9.52 ± 1.01 G/epoch), but differences were only significant for dinner (*p* < 0.001).

Pre-peak DST in breakfast ZT (29.37 ± 0.36 °C) showed a descending trajectory and became more stable after the glycemic excursion (28.84 ± 0.34 °C). In lunch ZT, this circadian index was clearly constant, in contrast to a clear ascending tendency in dinner ZT, with the post-peak temperature being significantly higher than that before glucose peak (28.84 ± 0.41 vs. 30.63 ± 0.98 °C; *p* < 0.001).

## 4. Discussion

This study analyzes the accuracy of self-declared eating patterns using an objective physiological variable: continuous glucose monitoring (CGM). Our results demonstrate that KronoEat, a smartphone-based application, could be a feasible tool for the self-recording of meal times, showing an adequate engagement rate and a high degree of concordance with significant peaks in interstitial glucose.

Although some studies using apps for meal pattern evaluation have shown valuable findings [40], accuracy in registration has not been evaluated. In our case, glycemic excursions of CGM data were used to estimate the accuracy of meal event annotations, using an already-validated algorithm for glucose peak detection based on the mean amplitude of glycemic excursions (MAGE) [36]. Although no consensus “gold standard” for meal detection has been established to date, continuous glucose monitoring seems quite promising in healthy populations [41]; therefore, postprandial glycemic excursions were compared with KE and KW events.

Overall results from KW (3.03 events/day/person) and KE (3.53 events/day/person) were realistic and coincident with the most common pattern of 3 meals/day in Spain [42]. However, a preference to use the app in comparison to the KW event marker button was noted. This preference may be related to the widespread use of smartphones in our daily life—for Spanish people, this reaches an average of 2.8 h per day, being around 30 min higher for people from 16 to 25 years old than for other age groups [43]. Nevertheless, the simultaneous use of KE and KW cannot be discarded because volunteers might not always prefer to use the application rather than KW, especially in circumstances where they do not have access to their mobile phones. In addition, one volunteer did not understand how to properly use the event marker button during recruitment, with the KE record thus being essential in this case. KE and KW event marker can therefore be used simultaneously to establish a synergistic relationship in eating pattern detection.

A recent review stated that there are few functional apps available to register eating schedules [44]. However, they are not problem-free. Most of them permit volunteers to edit food annotations and can include reminders or recommendations, which could bias final meal annotations (mixing actual and recall registrations) or alter habitual routines in observational studies relying on feedback. In addition, some apps can encounter privacy issues as they work with photo stamps [40] or give personal information to third parties [44]. KronoEat does not include the possibility to delete information, only food records are saved, and volunteers only have a calendar as feedback (see Figure S1). KE was designed specifically to study eating patterns, not caloric intake, and data are only used for research purposes.

The eating patterns registered in KronoEat showed a late window, which can be explained by a tendency towards later meal schedules in Spain compared to other European countries [45]. Surprisingly, snacks were the most registered event, addressing one of the main limitations of written questionnaires, which usually overlook the times at which snacks are ingested [46]. In fact, some volunteers practiced late-night snacking, as can be seen in Figure 3a, and measuring this data allows for a more accurate measurement of fasting periods. An interesting finding was that only a residual number of the feeding events recorded (6.1%) corresponded to recalls, revealing a preference in the participants for prospective annotation. Finally, breakfast was less frequently recorded by the participants, in line with current evidence [47], pointing to breakfast as the most skipped meal of day.

Glucose peak-related accuracy, a novel descriptor of eating time evaluation, has shown differences between distinct food events. In comparison to dinner and snacks, daytime main meals might produce a detectable glucose excursion more often, as lunch usually has a higher caloric content [48,49] and breakfast usually contains a higher percentage of sugars [50]. Indeed, in our study, after-dinner glycemic excursions had lower amplitudes than those related to breakfast and lunch. Nevertheless, this study demonstrated that average postprandial glucose levels were elevated after declared main meals, and the architecture of the corresponding glycemic excursions was characterized in *zeitgeber* time representations.

In addition, it has been suggested that CGM-only approaches may be of great interest since they only imply one single sensor, but multivariable recordings also hold great potential and should thus be investigated further [51]. Specifically, these authors point out that combining the strengths of different sensors (e.g., CGM and wristbands, as used here) may yield better results for meal detection, especially considering that possible masking factors such as activity [41] can be considered simultaneously.

This eating time protocol was designed as a multivariable approach, monitoring eating schedules jointly with the circadian status of participants. ACM measured variables, including MI and DST, also allow for the calculation of sleep periods in the participants. Thus, “metabolic time,” understood as the time of the metabolic fluctuations produced by energy intake, can be interpreted together with the other circadian descriptors to assess internal order impairment or chronodisruption. According to the significant changes observed in postprandial glucose in this study, feeding time, as measured by KE, has the potential to be an accurate indicator of carbohydrate metabolic time.

Sleep is a physiological process of relevance to metabolic homeostasis, which is altered in metabolic diseases [52]. Exploring the interaction of eating patterns with sleep quality is possible with the protocol proposed in this study. Moreover, CGM can be used to study the dynamics of glucose during sleep in prolonged ambulatory conditions, complementing other studies with shorter-duration approaches [53].

Furthermore, ACM—and, more specifically, movement intensity—provides relevant information with which to interpret glucose peaks since physical activity promotes glucose uptake by tissues, affecting the measurement, as already mentioned [54]. For example, Figure 5a shows that the physical activity increment during the morning is slightly delayed with respect to the corresponding glycemic excursions of breakfast, discarding the confounding effect of movement in our glucose peak characterization.

Finally, DST rhythm, which shows higher values during the night, might be of interest in meal timing studies because it is a potential marker of internal time. DST shows anticipation to sleep [55], as is the case in this study, with DST clearly increasing after dinner. The optimal eating window for humans is considered to be an early one [56], but concrete recommendations are still under debate. This methodology allows for the exploring of personalized approaches, relating dinner timing to DST nocturnal elevation as a marker of the melatonin phase [57]. This aspect might be relevant given the link between melatonin signaling and glucose metabolism, grounded in the relationship between impaired fasting glucose and genetic variations in the melatonin receptor [58], as well as amplitude changes in DST with respect to type 2 diabetes prognosis [59].

Our experiment is not free of limitations. Although physical activity was included as a possible confounding variable of interstitial glucose, there are a few factors affecting glycemic dynamics which were not measured in our protocol, such as the nutritional content of each food event. Also, measuring the duration of each food event was initially considered, but this was not implemented because of adherence reasons, constituting a further limitation of this study. However, tracking how much time people spent eating would have multiplied volunteer effort. The age of participants must also be considered, as young adults might be more likely to use smartphone applications than older generations. Moreover, it would have been interesting to interpret the effect of night meal events in relation to internal time and sleep, but circadian phases through DLMO and chronotypes were not measured. Again, nutrient profiling was not undertaken in order to minimize the subjects’ burden, but it could be useful in future studies focused on the effect of specific caloric intake. Finally, although the 1-week approach has some benefits (e.g., registering data from both working and free days), long-term adherence should be checked with further longitudinal or longer-duration studies.

## 5. Conclusions

To summarize, self-registered eating events via the KronoEat app were significantly correlated with postprandial glycemic dynamics under ambulatory conditions, demonstrating that data derived from continuous glucose monitoring are valuable as a means of measuring physiological accuracy in chrononutrition. Additionally, the simultaneous recording of sleep, activity, and distal skin temperature patterns, together with meal schedules, provides a comprehensive framework for future studies exploring metabolic and circadian timing under real-life conditions.

## 6. Patents

KronoEat is an informatics tool registered as a protected intellectual property in Spain (ID: 08/2025/1223). Authorship is shared equally between the University of Murcia and the Foundation for Health Research and Training in the Region of Murcia (FFIS).

## Data Availability

The data presented in this study are available on request from the corresponding author because it is an ongoing study.

## Supplementary Materials

The following supporting information can be downloaded at https://www.mdpi.com/article/10.3390/nu18050772/s1: Figure S1: KronoEat interface details.

## Abbreviations

The following abbreviations are used in this manuscript:

ACM	Ambulatory Circadian Monitoring
CGM	Continuous Glucose Monitoring
DST	Distal Skin Temperature
GLU	Glucose
KE	KronoEat
MAGE	Mean Amplitude of Glycemic Excursions
MI	Movement Intensity
SCN	Suprachiasmatic Nuclei
ZT	Zeitgeber Time
