# Supplementary material for: Evaluating the Accuracy of Declared Eating Schedules by Continuous Glucose Monitoring
Source: Nutrients. 2026 Feb 27;18(5):772. doi: 10.3390/nu18050772 (PMC12987204; doi:10.3390/nu18050772)
Supplement: Supplementary file 1 [file nutrients-18-00772-s001.zip › nutrients-4125458-supplementary.pdf]

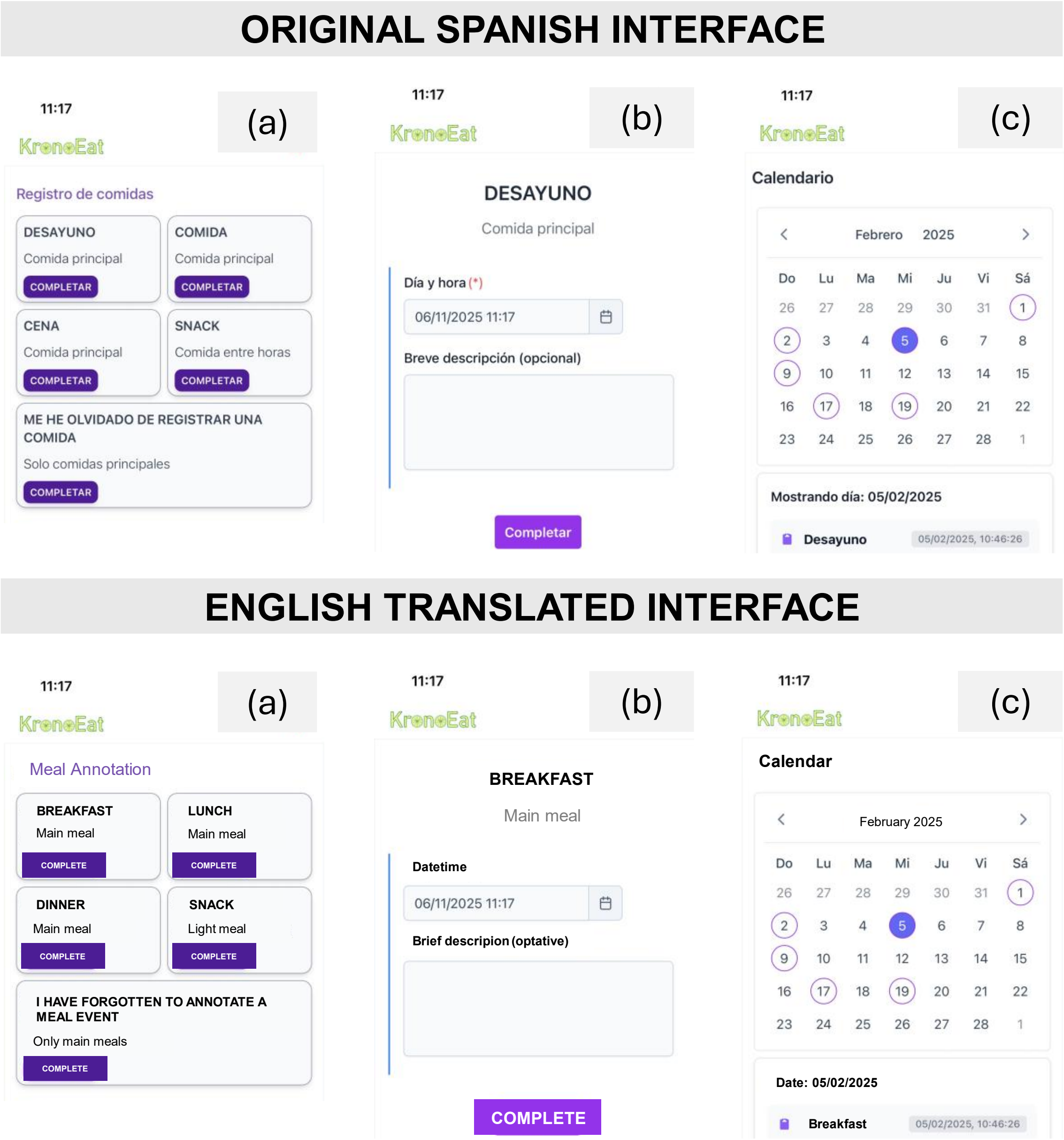

Figure S1. KronoEat is a digital tool to annotate meal schedules. (a) Volunteers have the possibility to record prospective data ("Breakfast", "Lunch", "Dinner" and "Snack") or recalls ("I have forgotten to annotate a meal event"; only for main meals). (b) After choosing a prospective option, time is synchronized from the device, and participants might add an optional brief description of food. Then, they confirm the annotation. For recalls, time is completed by the volunteer. (c) A calendar with marks for each annotation is offered for volunteers to provide them with feedback.
